# Discordant HIV Test Results: Implications on Perinatal and Haemotransfusion Screening for HIV Infection, Cape Coast, Ghana

**DOI:** 10.1155/2017/2857397

**Published:** 2017-10-08

**Authors:** Ato Kwamena Tetteh, Edward Agyarko

**Affiliations:** ^1^Laboratory Department, Metropolitan Hospital, P.O. Box 174, Cape Coast, Ghana; ^2^Department of Community Medicine and Health, Anglican University College of Technology, P.O. Box 74, Nkoranza, Ghana

## Abstract

Screening results of 488 pregnant women aged 15–44 years whose blood samples had been tested on-site, using First Response® HIV 1/2, and confirmed with INNO-LIA™ HIV I/II Score were used. Of this total, 178 were reactive (HIV I, 154; HIV II, 2; and HIV I and HIV II, 22). Of the 154 HIV I-reactive samples, 104 were confirmed to be HIV I-positive and 2 were confirmed to be HIV II-positive, while 48 were confirmed to be negative [false positive rate = 17.44% (13.56–21.32)]. The two HIV II samples submitted were confirmed to be negative with the confirmatory test. For the 22 HIV I and HIV II samples, 7 were confirmed to be HIV I-positive and 1 was confirmed to be HIV I- and HIV II-positive, while 14 were confirmed to be negative. Of the 310 nonreactive samples, 6 were confirmed to be HIV I-positive and 1 was confirmed to be HIV II-positive [false negative rate = 5.79% (1.63–8.38)], while 303 were negative. False negative outcomes will remain unconfirmed, with no management options for the client. False negative rate of 5.79% requires attention, as its resultant implications on control of HIV/AIDS could be dire.

## 1. Introduction

Practically, the only way children below 5 years of age in Ghana could be infected with the Human Immunodeficiency Virus (HIV) is through the mother-to-child mode or haemotransfusion. Without interventions to minimize vertical transmission, nearly 30% of infected expectant and breastfeeding mothers would add more children every year to those previously infected [1]. It is expected that up to 45% of children born to HIV positive mothers in low resourced countries will become infected [2]. Concurrent diagnosis of infection, antiretroviral therapy (ART), safer obstetric practice and infant feeding, counseling, and support can reduce the risk to <2% [3–5]. Interventions for prevention of vertical transmission of HIV were piloted in Ghana with support from UNICEF and other United Nation Agencies at Atua and St. Martin de Porres Hospital in Manya Krobo District, Eastern Region, from December 2001 [1]. There is currently an existing protocol in all health centres, polyclinics, district/municipal/metropolitan hospitals, and regional/teaching hospitals throughout Ghana which provides antenatal care (ANC) as well as prevention of mother-to-child transmission (PMTCT) interventions.

Recently, the Ministry of Health/Ghana Health Service has established that up to 95% of all pregnant women in Ghana make at least one ANC visit. By this, they access PMTCT for HIV interventions. Health facilities that provide PMTCT services engage the women and their families in a continuum of HIV prevention, care, and treatment services. The First Response HIV 1/2 test kit has been adopted for use as initial/screening test at the antenatal care units and also for blood safety throughout Ghana. For this test, if the control band alone appears, the test is considered “nonreactive” and therefore “negative.” The test result is concluded as “negative.” If the test band appears in addition to the control band, the test is reactive, and a supplementary test using a more sensitive rapid HIV test (OraQuick® HIV-1/2) is employed for confirmation of positivity. Initial tests offer the presumptive detection of antibody-positive specimens, and supplementary tests are used to validate whether specimens contain antibodies explicit to HIV. A reactive supplementary test result is indicative of a true positive result. In a case of indeterminate results between these two initial tests, a laboratory-based Enzyme-Linked Immunosorbent Assay (ELISA) test is performed at the Public Health Reference Laboratory, Korle-Bu, Accra, Ghana. This study presents evidence (from the 2010–2016 HIV Sentinel Survey Data) of the likelihood of obtaining false negative HIV test results using First Response HIV 1/2 alone at PMTCT centres and during blood donor screening.

Since 1992, the National HIV Prevalence Extrapolations have been derived from the HIV Sentinel Survey (HSS). This is a nationwide cross-sectional survey targeting pregnant women attending antenatal clinics in selected ANC sites in Ghana. Prenatal women have been the target of many seroprevalence studies because they are generally representative of all women of reproductive age, and they provide an accessible cohort for HIV testing and a secure sampling frame [6–8]. Data collection for sentinel surveys re through (urban/suburban) prenatal healthcare centres, considered sentinel sites, and nonrandom selection of sites can substantially affect results. This HSS is the primary data source for HIV prevalence in Ghana. It has been based on the principle that prevalence of HIV among pregnant women is a good proxy indicator of the spread of the infection among the general population [9]. The methodology and prevalence estimation are adequately explained in all editions of the HSS report.

The Metropolitan Hospital is one of 40 ANC sentinel sites, which draws blood samples for on-site preliminary First Response HIV 1/2 testing. Subsequently, plasma from individual samples is separated into 1.2 ml cryogenic vials and sent to the PHRL. After supplementary testing using immunoblot (INNO-LIA HIV I/II Score) at the PHRL, test results are returned to participating facilities. Results compiled over the past seven years at the Metropolitan Hospital, Cape Coast, are indicative of both false positive and false negative rates, which are biologically significant and could have dire consequences on infants and mothers. Of principal concern is the fact that women whose tests turn out to be negative at the PMTCT centres will go through the ANC without the appropriate intervention. Also, blood donors whose preliminary tests turn out to be negative will be bled and discharged. We recognize that interventions for PMTCT and blood donor screening in Ghana over the years have not included continuous reassessment for the fidelity of testing protocols. This article presents, “*prima facie,*” a retrospective analysis on the implications of probable false negative and false positive results, which could culminate from the sole use of First Response HIV 1/2 test at PMTCT centres and for blood donor screening.

## 2. Materials and Methods

### 2.1. Study Area

**Figure 1:**
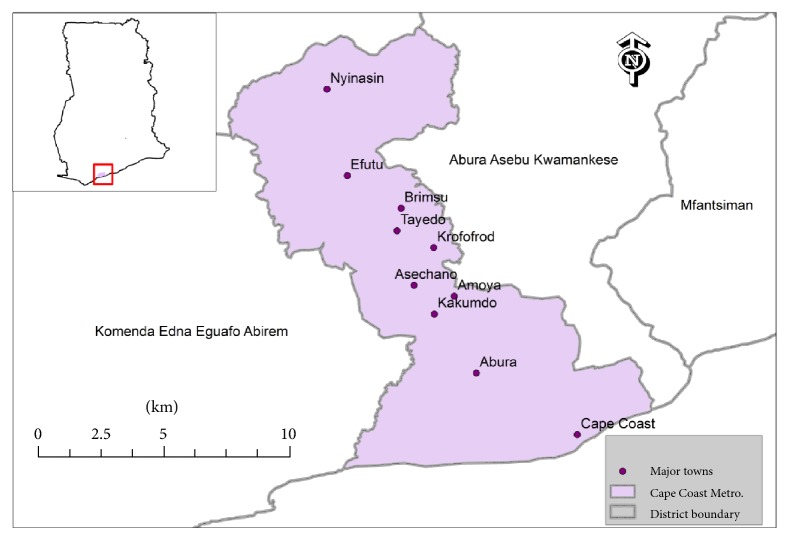
Map of Cape Coast Metropolitan Area* (Credit: Dr. Charles Gyamfi, Department of Civil Engineering, Tshwane University of Technology, Pretoria, South Africa)*.

The Cape Coast Metropolitan Area (Figure 1) is the capital of Central Region, Ghana. It is bounded on the south by the Gulf of Guinea, on the west by Komenda/Edina/Eguafo/Abirem Municipal, on the east by the Abura/Asebu/Kwamankese District, and on the north by the Twifo/Hemang/Lower Denkyira District (Figure 1). The metropolis covers an area of 122 km^2^ and it is located on longitude 1°15′W and latitude 5°06′N. The population of the metropolis according to 2010 population and housing census stands at 169,894 with 82,810 males and 87,084 females [10]. The Cape Coast metropolis experiences high temperatures throughout the year. The hottest months are February and March, just before the main rainy season, while the coolest months are June, July, and August. The metropolis has an annual rainfall total between 750 and 1,000 mm. The present vegetation of the metropolis consists of shrubs of about 1.5 m high, grasses, and a few scattered trees. The original vegetation of dense scrub, which the rainfall supported, has been replaced by secondary vegetation as a result of clearing for farming, charcoal burning, bush fires, and other human activities. Presently, trees are less dense in the area compared with the interior forest areas. Coastal dwellers are mostly fisher folks. The area is also considered an educational hub in Ghana, with some of the best colleges in Ghana.

### 2.2. Study Site

The Metropolitan Hospital is one of the forty nationwide sites participating yearly in Ghana's HSS. Over the past 24 years, the hospital has been able to consistently provide at least the minimum number of clients necessary to be included in the survey. The site also satisfies all criteria required for participation in the HIV Sentinel Survey (HSS), which are adequately listed in the 2015 HSS report [11]. To participate, one must be pregnant, must have an age between 15–49 years, and must be attending antenatal clinic for the first time during the current pregnancy within the survey period.

### 2.3. Population

The HSS survey targets all antenatal care (ANC) women aged between 15 and 49 years who were attending ANC clinic for the first time during their current pregnancy within the survey period. Pregnant women on repeat visit to the ANC during the period were excluded as set out by the HSS protocol. The expected sample size for each ANC site was 500, with an acceptable minimum of 251.

### 2.4. Study Design

This retrospective study made use of HIV Sentinel Survey data compiled over the last seven years at the Metropolitan Hospital. Data has already been used to estimate the national HIV prevalence, and data recording books returned to the Metropolitan Hospital. We found relevance in commenting on the discordant results realized in the past seven years in the testing of HIV among the pregnant women.

### 2.5. Ethical Consideration

Written informed consent was obtained from each study participant and the study was performed in accordance with the Declaration of Helsinki (1964).

### 2.6. Blood Sample Collection

Samples were taken from participants who met the eligibility and inclusion criteria set by the National AIDs/STI Control Programme. The closed system of blood draw (BD Vacutainer needles and evacuated 5 ml K_3_EDTA tubes) was used in obtaining approximately 3.5–4 ml of whole blood. Samples that were not tested on the same day were stored at 2–8°C and tested before the end of 72 hours.

### 2.7. On-Site HIV Testing

Samples were centrifuged to completely separate plasma from blood corpuscles. Preliminary HIV testing was done using the First Response HIV 1/2 test kits, as per manufacturer's instruction. Following this, the remaining plasma was transferred using Pasteur Pipettes into 1.2 ml cryogenic vials and labeled appropriately with participant site code, age, and test results.

### 2.8. Testing at the PHRL

Plasma (both reactive and nonreactive) was stored at −20°C till the end of the survey period and transported to the PHRL, where the confirmatory testing is done using immunoblot INNO-LIA HIV I/II Score. Throughout the period under study, all reactive samples had been confirmed by the PHRL. In addition, 10% of all nonreactive samples were randomly selected from the site and tested.

### 2.9. Data Analysis

All data that had been previously entered into a site register were recoded to seclude participants' identity and entered into Microsoft Excel®. This was later cleaned by removing test results that were not confirmed and subsequently exported into SPSS (Version 20.0: SPSS Inc., Chicago, IL), which was used for the analysis. Frequency distribution of age groups and crosstabulations for both tests were done. Summarily, the study is concentrated on the biological significance of probable clinical outcome of the results. The following parameters were also deduced to aid discussion:

(1) sensitivity=aa+c,specificity=db+d,false  positive  rate=bb+d,false  negative  rate=ca+c,

where *a* denotes true positive; *b* denotes false positive; *c* denotes false negative; *d* denotes true negative.

## 3. Results

### 3.1. Demographic Characteristics for This Study

A total of 488 participants who attended antenatal clinic at the Metropolitan Hospital within the survey period whose initial test results got confirmed were included in the following analysis. This number included all yearly reactive test samples and 10% randomly selected nonreactive samples. Participants ranged between 15 and 44 years (mean age: 26.09 years; mode: 23 years; standard deviation: 6.511 years). Majority of the participants were within the age group of 15–34 years. Participants who were ≥35 years old constituted the minority (Table 1).

### 3.2. Yearly Summaries of Samples Submitted

Over the past seven years, 3,245 plasma samples have been submitted by the Metropolitan Hospital to the PHRL for confirmation (Table 2). In total, preliminary on-site testing showed that 154 were HIV I-reactive and two were HIV II-reactive, while 22 were HIV I- and HIV II-reactive.

### 3.3. Seroprevalence of HIV Infection (2010–2016)

**Figure 2:**
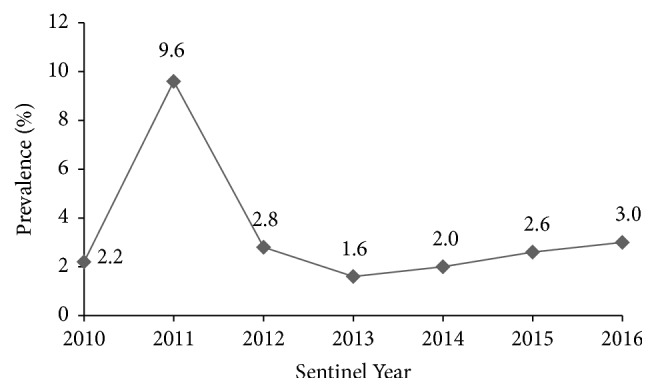
Metropolitan Hospital HIV prevalence trend (2010–2016).

Estimated prevalence for Cape Coast over the period was extracted from the 2016 HIV Sentinel Survey report and used to obtain Figure 2. With the exception of year 2011, which recorded the highest prevalence ever (9.6%) in the history of HIV in Cape Coast, the remaining were ≤3.0%. For all these years, our laboratory submitted the required number of samples necessary to participate in the Sentinel Survey.

### 3.4. Distribution of Serotypes among Age Groups

Of the 488 samples selected for confirmation over the seven-year period, 178 were reactive, while 310 were nonreactive (Table 3). Specifically, 154/178 were HIV I-reactive and 2 were HIV II-reactive, while 22 were HIV I- and HIV II-reactive. Out of 218 participants in the 15–24 years category, 81 were reactive for at least one of the different serotypes. Likewise, 69 and 27 participants in the age groups of 25–34 and 35–44 years, respectively, were reactive.

### 3.5. Crosstabulation of Preliminary and Confirmatory Tests

The more sensitive INNO-LIA HIV I/II Score was used as a standard against which sensitivity and specificity of the First Response HIV 1/2 were estimated (Table 4). After confirmatory testing, 121/488 were reactive, while 367 were nonreactive [sensitivity = 94.21% (90.06–98.37); specificity = 82.56% (78.68–86.44)]. Of the 154 HIV I-reactive samples submitted, 104 were confirmed to be HIV I-positive and 2 were confirmed to be HIV II-positive, while 48 were confirmed to be negative [false positive rate = 17.44% (13.56–21.32)]. The two HIV II samples submitted were confirmed to be negative with the confirmatory test. For the 22 HIV I and HIV II samples submitted, 7 were confirmed to be HIV I-positive and only one was confirmed to be HIV I- and HIV II-positive, while the remaining 14 were confirmed to be negative. Of the 310 nonreactive samples 6 were confirmed to be HIV I-positive and 1 was confirmed to be HIV II-positive [false negative rate = 5.79% (1.63–8.38)], while the remaining 303 were negative.

## 4. Discussion

HIV prevalence result of the 7-year period under study was ≤3.0% with the exception of the year 2011, which recorded the highest value of 9.6%. The Metropolitan Hospital where this prevalence was recorded drew attention nationwide. The Public Health Reference Laboratory retested all samples for 2011. Later, a general survey was conducted for the area before results for the year were accepted and included in the nationwide HIV Sentinel Survey. Reference is made to the March 2011 second civil war in Ivory Coast, where majority of Ghanaian residents returned to Ghana. Due to the civil unrest, a refugee camp called “Egyirkrom Refugee Camp” was set up by the Government of Ghana, at Egyirkrom, about 20 kilometers from Cape Coast. This camp hosted Ghanaians, Ivorians, and other Nationalities who came to the Central Region to seek refuge. Ghanaian returnees who reunited with families or got integrated into society and other refugees might have sought antenatal care between September and December 2011 at the Metropolitan Hospital, which serves as the first referral centre, and could probably have added to the existing prevalence. We decided not to conclude on this subtle explanation because it had come from a plenary discussion and not from scientific evidence. We would like to emphasize that this study rather focused on discordant outcomes detected at the Metropolitan Hospital (an HIV Sentinel Testing site), Cape Coast, Ghana.

The eventual consequence of misdiagnosing HIV infection in pregnant women and blood donors could be disastrous for Ghana and Africa as a whole, as it could frustrate strategic efforts aimed at controlling the spread. It is generally accepted that knowledge of HIV status is significant in increasing access to HIV treatment, care, and support in a timely manner. It grants people living with HIV an opportunity to receive information and tools to grossly minimize HIV transmission to others and also increases the impact of antiretroviral treatment (ART) [12]. Key policies are already under implementation to curb the rate of spread.

In all HIV testing services, it is essential that the results given to individuals be reliable. According to WHO recommendations, HIV testing methods should have sensitivity of at least 99% and specificity of at least 98%. This means that the test should give a positive result when the condition is present at least 99% of the time, and the test must also give a negative result at least 98% of the time when the patient does not have the condition [13]. We recognize that the most consistent screening methods, especially nucleic acid amplification testing technologies [14], which allow for very early detection, are costly and cannot be used routinely in low-resourced countries. It is, however, important that preliminary test kits be monitored consistently against more sensitive methods before they are used at testing centres such as the prevention of mother-to-child transmission (PMTCT) and donor screening laboratories. Without this, a good proportion of infected pregnant and breastfeeding women would add children to the pool of those already infected yearly [1], especially when most screened blood is given to pregnant women in Ghana.

In this study, the INNO-LIA HIV I/II Score test kit (sensitivity = 100%; specificity = 96.7) was used as a standard, against which the sensitivity of the First Response HIV1/2 was estimated. Sensitivity and specificity of 94.21 and 82.56%, respectively, were estimated for the period under review. We suggest the use of a parallel algorithm which tests all specimens with two rapid tests and resolve discordant results by retesting with a third, tiebreaker test [12] in the near future, especially for pregnant women and blood donors

From the results, 121 out of the 178 positive results (representing 68%) were confirmed to be positive. Thus, 32% of the samples were false positive (false positive rate = 17.44; CI: 13.56–21.32). With the existing testing protocol for HIV in Ghana, all false positive outcomes will be confirmed with OraQuick HIV-1/2 just as true positive outcomes at the PMTCT centres, while blood donors will fail outright and will be directed to the designated counseling units for further attention. With this, the popular issues related to stigma and discrimination, attendant psychological and social consequences, domestic violence, and exposure to the unnecessary and potentially toxic medical treatment are avoided to some extent.

This study detected false negative outcomes. Out of the total, 2.3% of the nonreactive samples turned out to be false negative (false negative rate = 5.79% (1.63–8.38)). Although this rate looks small in percentage terms, it has a major biological significance in terms of the eventual consequence. False negative outcomes are a threat not only to public health prevention strategies but also to the health and well-being of the individuals. A false negative diagnosis, despite being incorrect, may prevent patients from seeking out other testing opportunities, taking the necessary precautions to prevent the transmission of HIV, and receiving the care and treatment that they need [15]. Without the necessary obstetric precautions and ART in pregnant women who are falsely diagnosed, there is an increased chance of perinatal transmission.

## 5. Conclusion

We would like to emphasize that it is important to maintain the integrity of PMTCT and blood donor testing of HIV by ensuring the reliability of the testing methods. In order to improve detection of HIV, we suggest that efforts should be made to always provide more sensitive supplementary testing no matter the outcome of the preliminary test at the PMTCT centres and also during donor blood screening.

## Figures and Tables

**Table 1:** Age ranges of participants.

Age range (years)	Number of participants (%)
15–24	218 (44.7)
25–34	205 (42.0)
35–44	65 (13.3)

*Total*	*488*

**Table 2:** Summaries of samples collected and results obtained.

Year	Number of samples collected	Test results (First Response HIV 1/2)		
		HIV I	HIV II	HIV I and HIV II
2010	500	25	—	14
2011	500	64	—	3
2012	500	19	—	2
2013	500	12	1	1
2014	391	10	1	2
2015	389	10	—	—
2016	465	14	—	—

**Table 3:** Prevalence of serotypes among age groups.

Age (years)	Reactive (First Response HIV 1/2)			Nonreactive	Total
	HIV I	HIV II	HIV I and HIV II		
15–24	71	2	8	137	218
25–34	61	0	8	136	205
35–44	22	0	6	37	65

*Total*	*154*	*2*	*22*	*310*	*488*

**Table 4:** Crosstabulation of initial and supplementary tests.

First Response HIV 1/2	INNO-LIA HIV I/II Score				Total
	HIV I	HIV II	HIV I and HIV II	NR^*∗*^	
HIV I	104	2	—	48	154
HIV II	—	—	—	2	2
HIV I and HIV II	7	—	1	14	22
NR^*∗*^	*6*	*1*	—	303	310

*Total*	*117*	*3*	*1*	*367*	*488*

NR^*∗*^: nonreactive. Sensitivity = 94.21% (90.06–98.37); specificity = 82.56% (78.68–86.44); false positive rate = 17.44% (13.56–21.32); false negative rate = 5.79% (1.63–8.38).
